# Creative thinking in Parkinson’s disease: A systematic review and meta-analysis

**DOI:** 10.1007/s10072-026-09234-7

**Published:** 2026-07-16

**Authors:** Laura Colautti, Sara Magenes, Francesco Pagnini, Maria Caterina Silveri, Anna Rita Giovagnoli, Alessandro Antonietti

**Affiliations:** 1 Department of Psychology, Università Cattolica del Sacro Cuore, Largo A. Gemelli, 1, 20123 Milan, Italy; 2 Fraternità E Amicizia Società Cooperativa Sociale ONLUS, Milan, Italy; 3 Fondazione IRCCS Istituto Neurologico Carlo Besta, Milan, Italy; 4 IRCCS Fondazione Don Carlo Gnocchi, Milan, Italy

**Keywords:** Parkinson’s disease, Creativity, Divergent thinking, Dopamine, Executive functions, Cognition

## Abstract

**Introduction:**

Creativity in Parkinson's disease (PD) has aroused research interest due to its neurological underpinnings, which involve brain regions crucial for creative and divergent thinking (a core-process of creative thinking), and because of some patients who displayed an increased artistic drive following dopaminergic treatment. From a cognitive point of view, creative thinking underlies several cognitive abilities, such as executive functions and memory. Therefore, a better understanding of whether it is increased (or, at least, preserved) in PD patients can provide useful insights for sustaining cognitive functioning. The aim of the present study was to investigate whether PD patients regularly assuming dopaminergic medications present higher divergent thinking skills than healthy controls.

**Methods:**

A meta-analysis was conducted according to the PRISMA guidelines to provide a statistical synthesis of the studies. Study quality was assessed using the QUADAS -2 tool.

**Results:**

Ten studies were included in the meta-analysis, which indicated the absence of significant differences between PD patients and healthy controls in tasks assessing divergent thinking (Cohen’s d = -0,095 (95% CI: -0,308, 0,118)).

**Conclusions:**

Such findings support the notion that divergent thinking can be spared by the disease, maybe constituting a possible resource for patients’ cognitive functioning, as it involves those cognitive abilities that can compensate impairments in PD. Clinical implications and guidance to further studies and interventions to support PD patients’ cognition were discussed.

**Supplementary Information:**

The online version contains supplementary material available at 10.1007/s10072-026-09234-7.

## Introduction

### Parkinson’s disease

Parkinson’s disease (PD) is a slow-progressing neurodegenerative disease, mainly due to the presence of α-synuclein insoluble aggregates neural inclusions in the form of Lewy bodies and a loss of dopaminergic neurons in substantia nigra pars compacta in the mesencephalon, a core component of the basal ganglia [1]. As the disease progresses, other cerebral structures are involved, affecting both subcortical and cortical regions [2, 3]. While primarily known for its motor symptoms – such as bradykinesia, rigidity, tremor, and gait disturbances [4] – PD also involves widespread non-motor symptoms, including cognitive impairments.

Focusing on cognition, selective cognitive difficulties can be present till the early stages of the disease. A particular attention deserves executive functions, which are frequently impaired in PD and are pivotal for daily activities [5]. They encompass various components including inhibition, updating, planning, initiation, monitoring of an action, and cognitive flexibility [6, 7]. Other abilities can be related to them, such as working memory and verbal fluency [6, 8], and are involved in the correct execution of cognitive operations, such as visuospatial skills [9], the retrieval of information [10], and decision making [11], that can be impaired in PD as well with detrimental consequences [12–15]. As the disease progresses, cognitive impairments can worsen and be a risk factor for the reduction of personal autonomy and the development of dementia as well [16].

### Creative thinking

Creative thinking has been described as a complex ability, highly functional from a cognitive point of view, as it allows the individual to move away from habitual and automatic responses developing alternative behaviors that are appropriate for the contingent situation [17]. It serves an adaptive function in everyday life, supporting both enhanced problem-solving abilities through the assessment of available resources and the individual autonomy and wellbeing during the lifespan [18–21]. To do so, two core processes are present in creative thinking: divergent and convergent thinking. Divergent thinking, which is crucial for innovative and original ideas, can be described as the ability to find and produce more solutions or responses for an open-ended problem or question, underlying the ability to think in different directions and combine different domains. Convergent thinking entails the finding of a single correct solution to a clearly defined problem, underlying the ability to reorganize and connect existing knowledge in a new way [22].

There is evidence that, during the generation of creative ideas, several cognitive abilities are involved, encompassing executive functions, essential for performing a goal-oriented and appropriate cognitive process (including inhibition, flexible attentional changes, and the ability to maintain and manipulate information), and semantic and autobiographical memory, providing the retrieval of useful material for new ideas and (remote) associative links [23–25].

From a neuroanatomical level, it is well documented an overlap of neural areas involved in tasks assessing creative thinking (and in particular divergent thinking) and in tests that assess other cognitive abilities such as executive functions, where the prefrontal cortex (PFC) has a paramount role (e.g., [26, 27]) together with wide cortical and subcortical networks [28–30]. Consistently, according to the dual pathway model, a creative idea (characterized by both originality and appropriateness) mainly involves a dynamic interplay between cognitive flexibility, which enables the individual to shift from different mental sets and explore alternative perspectives, and persistence, which allows focusing in-depth on exploring a specific issue and methodically searching for solutions and inhibiting distractions (e.g., [31]). In this way, particular attention deserves the activations in the striatum and the PFC that are assumed to be respectively associated with flexibility and persistence (for more details, see: [32]). For an optimal creative process, these two cognitive processes have to work synergically (conversely, excessive flexibility without persistence can lead to distractibility and bizarre ideas, while rigid persistence results in inflexible and perseverative thinking). Dopamine may regulate such a balance, possibly through the dopaminergic pathways, by which dopamine is projected to striatal regions and PFC [32, 33]. This neurotransmitter is the one having the strongest influence on creativity [34], being also crucial for the creative drive, motivation, and process, modulating reward processing and supporting curiosity and exploration behaviors, remote mental associations, and flexible processing of information [22, 33].

### Creative thinking and Parkinson’s disease

On such a ground, over the last 20 years creativity has attracted interest from clinicians and researchers, both due to the pathophysiological peculiarities of PD – mainly involving structures pivotal for creativity (such as basal ganglia, frontal lobes, corticostriatal circuits, and dopamine pathways) – and for studies that showed that, after starting the dopaminergic treatment, some PD patients display the onset of a creative drive or an increase in (and sometimes an urge towards) the artistic activity, as it seems that their talent “seemed to flourish” following the initiation of the therapy [35]. In this way, several single-case reports documented the emergence or increase of artistic expression in PD patients following dopaminergic treatment. For instance, one patient began producing and selling artwork extensively after a dopamine agonist dose increase [36]. Another, a 47-year-old amateur painter, renewed and intensified his artistic activity one month after starting dopaminergic therapy, also gaining a certain degree of commercial success [37]. A third example involved a 55-year-old man who began writing high-quality poetry for the first time after starting medication, despite no prior experience [38].

In the attempt to provide possible explanations for such a change in behavior, several studies have hypothesized that increased levels of dopamine in the dopaminergic pathways, due to dopaminergic therapy, can be related to a reduction in latent inhibition, which is the ability to filter irrelevant stimuli from awareness. Such a reduction can sustain a more flexible information processing, make available more remote associations and the entrance into working memory of irrelevant concepts, and support the original recombination of ideas [32, 34, 39]. Accordingly, there is evidence of an association between low latent inhibition and high creativity (e.g., [40]). Moreover, through the dopaminergic pathways, dopamine can promote the cognitive processes underlying creative thinking, such as flexibility and persistence, but also reward-based drives and motivational processes. This is in line with the assumptions that link the increased artistic engagement displayed in PD patients after the beginning of the dopaminergic therapy, and especially after the intake of dopamine agonists, with the overstimulation of the mesolimbic dopaminergic pathways (e.g., [32, 33, 41]). However, it is still an open question whether the frequent engagement in artistic activities after starting dopaminergic therapy, especially dopamine agonists, can be considered an impulse control disorder (ICD), even though, unlike other ICDs, the increased creativity generally does not lead to negative or harmful effects on patients and their families [35, 37, 38].

Furthermore, it is unclear to what extent the increased artistic tendencies observed in single-case reports can be mirrored in a general increase in creative thinking (and the underlying cognitive processes) in patients treated with dopaminergic medications: The present paper focused attention on this issue. Since from a neuropsychological point of view creative thinking involves many cognitive abilities as important as vulnerable in PD patients, it appears important to investigate whether PD patients can present higher levels of creative thinking, also to better understand whether and how it can maybe support cognitive functioning in such patients preventing possible cognitive impairments.

To do so, it appears important to focus on cross-sectional design studies that assessed creative thinking through cognitive tasks that provide an objective measure, as there is a difference between creating something that has an original and useful value (underlying creative thinking) and the simple drive of creating something by engaging in an artistic activity [42, 43]. In this way, considering the scores obtained in divergent-thinking tasks can be functional, as divergent thinking is widely considered a core process of creativity [44] and a proxy of creative potential [45]. For instance, one of the most used tools to assess divergent thinking is the Alternate (or Alternative) Uses Task (AUT) [45], in which the individual has to produce as many different and uncommon uses for daily objects (e.g., a newspaper, a brick) as possible. There are also tasks using visual stimuli, always requiring finding as many answers as possible. The parameters for scoring the answers of most divergent-thinking tasks generally are: fluency (the number of appropriate answers produced), flexibility (the number of different categories the answers belong to), originality (the rarity of the answers compared to other participants or a normative group), and elaboration (the level of detail in the produced answers).

Given the conflicting findings reported in the literature comparing PD patients and healthy controls on divergent-thinking tasks, a meta-analysis that deepens such studies is useful, providing a statistical synthesis of the data. In particular, the present review was aimed at verifying the hypothesis that PD patients on a regular regimen of dopaminergic medications display higher levels of creative thinking, assessed through divergent-thinking tasks.

### Aim

The aim of the present study was to delve into divergent thinking in PD patients by investigating possible differences compared to healthy controls, and in particular whether PD patients present higher levels of divergent thinking. Specifically, the main research question was: Do PD patients regularly assuming dopaminergic medications present higher levels of divergent thinking than healthy controls?

## Methods

### Search strategies

The present review and meta-analysis was led according to the Preferred Reporting Items for Systematic Review and Meta-Analyses (PRISMA) [46]. The review protocol was registered in the PROSPERO registration (registration no. BLINDED FOR REVIEW). The search was updated on 11 September 2025. It included articles published since 2000 in peer-reviewed journals indexed in PubMed, Scopus, and PsycInfo. Preprints were also searched in MetaArXiv.

According to the aims, for PubMed, both Medical Subject Headings (MeSH descriptors, 2025 edition) and free-text terms were used, to maximize retrieval of relevant studies. The MeSH terms included “Parkinson Disease” and “Creativity”, which also covers related entry terms such as “Creative Thinking” and “Creative Ability”. To ensure completeness, additional free-text keywords such as “divergent thinking”[tiab] were added. For Scopus, PsycInfo, and MetaArXiv, the following terms were combined using Boolean operators: ("Parkinson’s disease" OR "Parkinson disease") AND ("creativity" OR "creative thinking" OR "divergent thinking" OR creativ*).

The inclusion criteria for deciding which studies to keep were: 1) the enrollment of samples of patients affected by primary Parkinson’s disease, according to UK Brain Bank criteria [47] and the presence of healthy control groups with which comparing patients’ divergent-thinking performances; 2) the assessment of divergent thinking through verbal or figurative stimuli, in which it is required to find more than one solution, where is present at least one of the parameters used to score them (i.e., fluidity, flexibility, originality, and elaboration; Accordingly, studies relying exclusively on self-report questionnaires or subjective measures of creativity were not included); 3) the evaluation of PD patients during the pharmacological “on” condition to limit possible variability related to dopaminergic status.

The exclusion criteria adopted were: 1) participants presented other neurological comorbidities; 2) participants presented a diagnosis of dementia; 3) participants underwent cognitive training specifically addressed to foster creative thinking; 4) book chapters or single-cases reports; 5) studies in which information to compute the effect size was not reported or was not possible to trace such data.

### Study selection

**Fig. 1:**
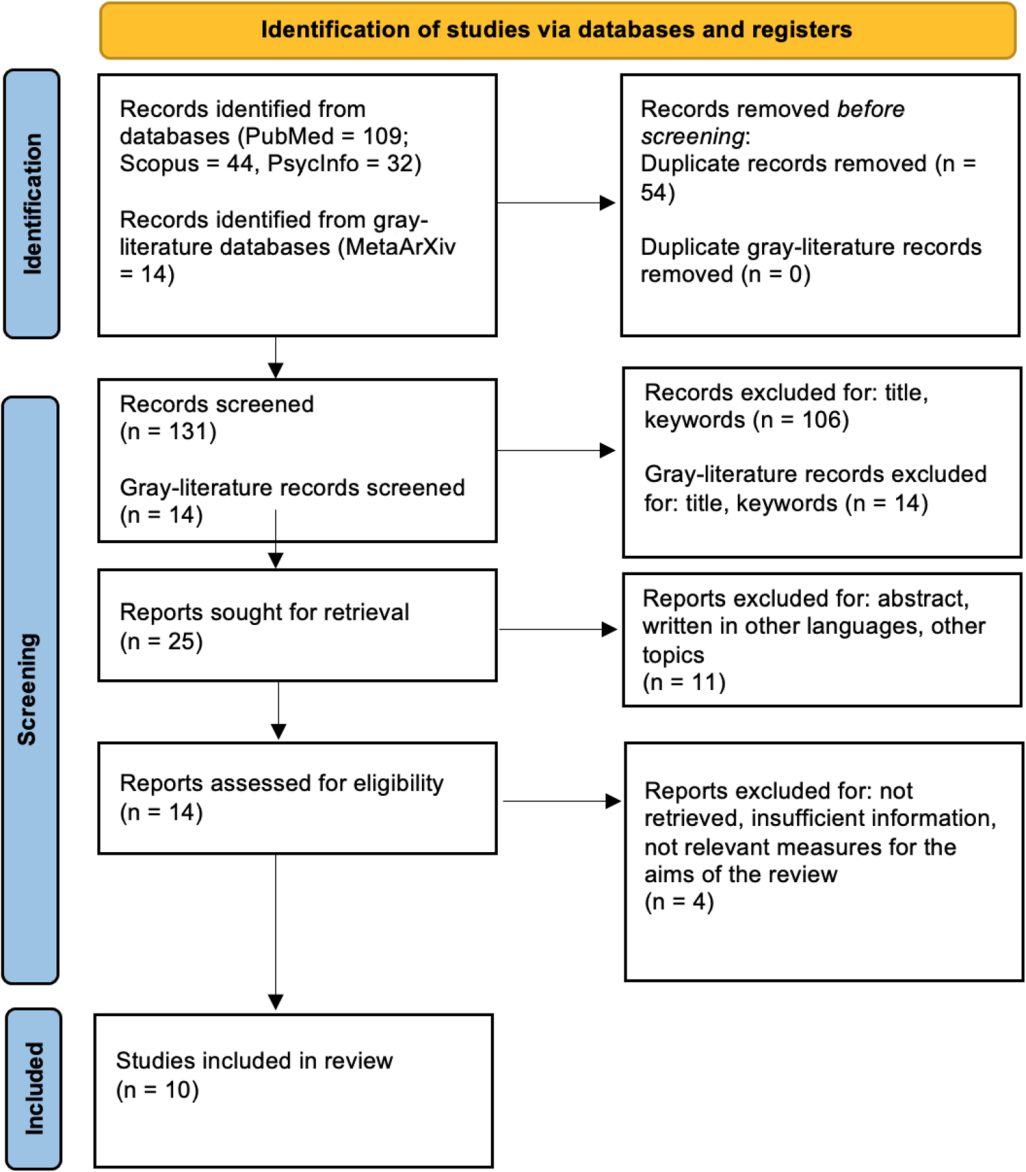
PRISMA flow diagram for the considered studies

L.C. and S.M. independently screened the relevant articles, first by title, keywords, and language and then by reading the abstracts and full texts. After the selection of the studies, bibliographies of the selected studies were checked to include other possible eligible studies. The selection of studies followed the PRISMA Statement [46] (see Fig. 1 for more details). Possible doubts about the inclusion of the studies were discussed with F.P. to reach a consensus.

### Data extraction and quality assessment

An electronic data collection sheet was designed to decide which variables to consider (e.g., authors and year of publication, country where the study was conducted, size of the sample of patients and controls, mean and standard deviations of the parameters of divergent thinking tasks, and, only for patients, clinical information such as the daily dosage of dopaminergic drugs). The corresponding authors’ email addresses were extracted, for requiring possible additional data if necessary.

The methodological quality and potential bias of the studies were assessed using the Quality Assessment of Diagnostic Accuracy Studies Instrument (QUADAS-2; [48]). The QUADAS-2 represents a structured tool that assesses the potential bias in four domains: patient selection (i.e., consecutive or random sample of patients enrolled), index test (how a study was conducted and interpreted), reference standard (how the target condition is classified), and flow and timing (the time interval and any interventions between index test(s) and reference standard). Two investigators (L.C. and S.M.) independently extracted data and evaluated study quality; Possible doubts were discussed with F.P.

Inter-rater agreement for study selection (Cohen’s kappa = 0.90), data extraction (Cohen’s kappa = 0.90), and risk-of-bias analysis (Cohen’s kappa = 0.88) was acceptable with almost perfect agreement [49].

### Analyses

Analyses were computed using Comprehensive Meta-Analysis Version 4.

Standardized differences in means (estimated by Cohen’s d) with 95% confidence interval (CI) were calculated only for those studies in which the means and the standard deviations were considered as the effect size for outcomes. Specifically, Cohen’s d – usually adopted for quantifying the magnitude of the difference between two groups [50] – was computed using the means and the standard deviations of the clinical group and the control one in the divergent-thinking tasks scores (i.e., fluency, flexibility, originality, and elaboration); Otherwise, we used the difference in means and p values derived from the difference of the two groups’ means in the tasks. The mean effect size was then computed by pooling the studies’ effect sizes with a random-effects model as the selected studies were not identical (e.g., in terms of study design or patients’ characteristics) [51].

To assess heterogeneity among the studies, the Q statistic was examined. When Q is significant, it highlights that the variance in the population of effect sizes is higher than expected from the sampling error. Tau-squared (*τ*^*2*^), the variance of true effect sizes, was also reported. I^2^ was also considered to investigate which proportion of the variance in observed effects reflects variance in true effects rather than sampling error.

Sensitivity analyses encompassed the assessment of the influence of each study on the overall estimates by recalculating the pooled outcome proportions when one study was removed and all others included.

Further analyses were computed to examine important study moderators. Subgroup analyses were launched to investigate possible differences in results according to the divergent-thinking parameters. One continuous moderator was considered as well (i.e., total Levodopa equivalent daily dose (LEDD) when studies reported it) with a meta-regression technique.

To assess publication bias, a funnel plot was considered.

## Results

### Study description

Ten studies met the inclusion criteria and were considered, denoting a great variability among them for the parameters considered to score the divergent-thinking tasks and the tools used, the study design, and the characteristics of the clinical samples. Where a study considered more parameters (among fluency, flexibility, elaboration, originality), for each of them a different line (constituting a case) was filled in the dataset, resulting in a total of 37 cases. Specifically, 11 cases considered fluency, nine flexibility, seven elaboration, and ten originality. See Supplementary Table 1 (Online Resource) for further information about the extracted and considered data for the analyses and the examined cases.

Other parameters, used only by a limited number of studies to investigate the performance, were not considered for the analyses. For instance, in the Abbreviated Torrance Test for Adults (ATTA) the “Creativity index” was used only by Drago and colleagues [52], since it derived from the sum of the four traditional parameters, each converted from raw scores to scaled scores through specific tables and then summed to a series of fifteen creativity indicators; For further information, see: [53]. As well, the “ATTA total”, used in two studies by Canesi and colleagues [42, 54], was not considered because it was obtained by summing the scores regarding the four traditional parameters. The “T factor” index from the DTT task [55], refers to the ability to produce relevant titles for every drawing made but it was discarded because it was used only by Ruggiero and colleagues [55].

Focusing on divergent-thinking tasks, four studies [43, 56–58] measured verbal divergent thinking using the AUT [45]. In this task, participants are prompted to list as many alternative uses – that have to be original and appropriate – as they can for a common object. For this study, common objects (e.g., spoon, brick, newspaper, rubber band, sheet, sock) were presented. A subjective scoring system is often employed, using raters blind to participants’ group assignment (following the methods of Hass & Beaty [59]). The analysis generally includes the calculation of the four scores: fluency, flexibility, originality, and elaboration. One study [43] used the Tel Aviv University Creativity Test (TACT; [60]), designed to evaluate different aspects of divergent thinking. The TACT version used by the authors includes two open-ended subtests: one verbal (Alternative Uses) and one visual (Line Meanings). The scoring of these tests is based on two key metrics: fluency (the number of responses) and quality (that is the originality of the responses). One study [55] analyzed divergent thinking through DTT [55, 61], which consists of 12 frames, featuring a simple graphic line that is used as a stimulus. Participants are instructed to use each line as a starting point to produce a drawing that is as original as possible. The evaluation of the designs is based on the four-factor scores of divergent thinking, as derived from Guilford's studies (i.e., fluency, flexibility, originality, elaboration) and a fifth factor consisting of scoring the title given for each frame. Three studies [42, 52, 54] evaluated divergent thinking using the ATTA [53]. The test includes three tasks that the subject is instructed to complete within a specified time frame. The ATTA provides measures of both verbal (finding as many consequences as possible of an unrealistic scenario) and visual creative abilities (producing original and detailed drawings). In addition, the visual tasks require the patients to label their drawings. Two studies [39, 62] included subtests derived from the Torrance Tests of Creative Thinking (TTCT; [63]) to measure divergent thinking, where scores are based on the four parameters of fluency, flexibility, originality, and elaboration. Specifically, in Canesi and colleagues’ [39] study, divergent thinking is examined through three activities of the TTCT that combine indices of verbal and visual creativity, similarly to the ATTA; In Polner and colleagues’ [62] study, the “Just Suppose” subtest of the TTCT was used to examine divergent thinking in the verbal domain, requiring to find as many ideas as possible of an unrealistic scenario.

Regarding the clinical samples, Canesi and colleagues [39] recruited the largest group (composed of 36 PD patients) and Varrone and colleagues [56] considered the smallest one (composed of 10 PD patients). The sample with the lowest mean age belongs to Salvi and colleagues’ (2021) study, while the highest to Drago and colleagues’ [52] one. Regarding the duration of the disease, Polner and colleagues [62] considered newly diagnosed patients, while the highest mean duration of PD emerged in Canesi and colleagues’ [39, 54] studies, which respectively present also the highest mean LEDD and the highest mean score for severity of the disease.

Five studies were conducted in Italy, and the others were equally distributed in Germany, Hungary, Israel, Sweden, and USA (see Table 1 for more details). Table 2 reported the detailed characteristics of the studies and the main results related to divergent-thinking performances.

**Table 1:** Characteristics of the samples

	*Drago* et al*., 2009* [52]	*Canesi* et al*., 2012* [39]	*Faust-Socher* et al*., 2014* [43]	*Varrone* et al*., 2015* [56]	*Polner* et al*., 2015* [62]	*Canesi* et al*., 2017* [54]	*Canesi* et al*., 2016* [42]	*Ruggiero* et al*., 2019* [55]	*Salvi* et al*., 2021* [57]	*Heldmann *et al*., 2024* [58]
Country	USA	Italy	Israel	Sweden	Hungary	Italy	Italy	Italy	Italy	Germany
Sample size PD (female%)	15 (0%)	36 (na)	27 (62.96%)	10 (20%)	18 (38.88%)	13 (46.13)	A: 12 (41.67%); NA: 12 (41.67%)	17 (23.53%)	13 (30.77)	20 (50%)
Sample size HC (female%)	7 (85.71%)	36 (na)	27 (44.44%)	12 (41.67%)	19 (36.84%)	13 (46.13)	A:12 (41.67%); NA: 12 (41.67%)	15 (40%)	24 (54.17)	21 (52.38)
Age PD: mean (SD)*	70.75 (9.69)	61 (7.5)	62 (7)	65 (8)	48.0 (7.2)	65 (11.9)	A: 53.7 (10.4); NA: 57.5 (9.2)	61.47 (9.6)	56.5 (9)	68.1 (9.9)
Age HC: mean (SD)*	69 (17.58)	60.2 (9.7)	59 (9)	65 (7)	47.6 (7.7)	66 (6.2)	A: 53.4 (8.8); NA: 56.9 (10.7)	61.87 (8.55)	61.3 (7)	67.3 (7.3)
Education PD: mean (SD)*	12.4 (3.69)	11.7 (4.8)	16 (3)	11 (3)	11.6 (3.3)	10.5 (5.1)	A: 13.7 (3.8); NA: 11.5 (3.9)	12.24 (4)	12.6 (3.8)	12.2 (2.9)
Education HC: mean (SD)*	13.79 (2.23)	9.5 (4.1)	17 (3)	12 (3)	11.8 (3.8)	10.5 (3.6)	A: 14.1 (4.3); NA: 11.4 (4.5)	11.33 (2.71)	14.1 (2.8)	13.0 (3.0)
Disease duration: mean (SD)*	8.08 (6.06)	9.7 (5.2)	5.8 (3.9)	6.4 (3.1)	newly diagnosed	9.6 (6.4)	A: 7.1 (3.4); NA: 7.1 (3.8)		8.7 (3.9)	7.15 (5.5)
UPDRS III: mean (SD)		19.4 (8)	24 (9)	12.8 (2.5)	-	34.7 (8.8)	A: 16.7 (11.6); NA: 18.3 (8.8)			18.1 (10.9)
Hoehn & Yahr: mean (SD)		2.1 (0.4)		1.7 (0.4)	-	3.1 (0.3)	A: 1.9 (0.5); NA: 2.3 (0.3)			
LEDD: mean (SD)		650 (222.6)	481 (430)	615.2 (272.8)	-	468 (257)	A: 410 (304); NA: 359 (201)	1456.735 (2277.8)		570 (311)

^*^ in years

blank: not reported

PD: Parkinson’s Disease group; HC: healthy control group; UPDRS III: Unified Parkinson's Disease Rating Scale – motor section; LEDD: Levodopa-equivalent daily dose; A: artists; NA: non artists; na: not available

**Table 2:** Features of the studies and main results related to divergent-thinking performances

Study	Inclusion criteria	Divergent thinking measures	Results
Drago et al., 2009 [52]	No history of head injury, psychiatric illness, or neurological illness PD patients: treated with dopaminergic medications; being in “on” condition during the assessment	ATTA (Creativity Index, fluency, flexibility, elaboration, originality)	No significant differences between PD patients and HCs in any of the ATTA scores
Canesi et al., 2012 [39]	Stable therapy with L-Dopa or DA for ≥ 4 months; tests scores were within the normal range; not have had artistic hobbies or professions before the diagnosis of PD	TTCT—three activities (fluency, flexibility, elaboration, originality)	No differences between PD patients and HCs in any of the TTCT scores, except flexibility (which was lower in PD patients than in HCs)
Faust-Socher et al., 2014 [43]	Therapy with L-Dopa or DA; graduation from high school; no history of brain surgery or DBS, or dementia	TACT—alternative uses and line meaning (fluency, originality)	PD patients performed better than HCs in TACT-Visual (both fluency and originality)
Varrone et al., 2015 [56]	Not having any psychiatric condition	AUT (fluency)	PD patients presented significant lower fluency than HCs
Polner et al., 2015 [62]	Newly diagnosed; assessment after 12 weeks of therapy	TTCT—Just suppose subtest (fluency, flexibility, originality)	No significant differences in the parameters of the TTCT subtest
Canesi et al., 2017 [54]	Absence of severe dysexecutive syndrome, severe dysarthria, severe bradykinesia; not undergone on antipsychotic nor antidepressant therapy; no history of impulsive-compulsive behavior	ATTA (total, fluency, flexibility, elaboration, originality)	No significant differences between PD patients and HCs in any of the ATTA scores
Canesi et al., 2016 [42]	MiniMental State Examination > 24; stable dopaminergic therapy for ≥ 8 weeks; not undergone on antipsychotic or anti-depressive therapy; no DBS	ATTA (total, fluency, flexibility, elaboration, originality)	ATTA total score was significantly higher in HC artists and PD artists. PD non artists had the lowest ATTA total score, but the difference with HC non artists was not significant
Ruggiero et al., 2019 [55]	Age between 45 and 85 years; age at onset of the disease > 40 years; MiniMental State Examination score ≥ 20; Hoehn and Yahr stage 1 to 3; absence of psychiatric illnesses, or history of drug abuse or alcoholism, diabetes mellitus, or cerebral infarction or tumour; PD therapy with L-Dopa or DA	DTT visual (total, fluency, flexibility, originality, processing, titles)	PD patients showed higher DTT total score and originality than HCs
Salvi et al., 2021 [57]	Absence of dementia; stable dopaminergic therapy for ≥ 4 months	AUT (fluency, flexibility, originality, elaboration)	HCs showed higher flexibility than the PD group; no other significant difference emerged
Heldmann et al., 2024 [58]	Absence of dementia; lack of clinical depression PD patients: treated with dopaminergic medications; being in “on” condition during the assessment	AUT (fluency, flexibility, originality)	Significant results emerged in the number of correct answers and in originality, where HCs showed higher scores than the PD group

ATTA: Abbreviated Torrance Test for Adults; AUT: Alternative uses task; DTT: Divergent Thinking Test; DA: dopamine agonist; DBS: deep brain stimulation; HCs: healthy controls; L-Dopa: Levodopa; PD: Parkinson’s Disease; RAT: Remote Association Test; TACT: Tel Aviv University Creativity Test; TTCT: Torrance Test of Creative Thinking

### Quality assessment

The quality of the studies was assessed with the QUADAS-2 tool (see Supplementary Table 2 for further details).

Studies were not excluded based on their risk of bias or applicability concerns. All studies had concerns in at least one of the domains (patient selection, index test, reference standard, and flow and timing). Regarding patient selection risk of bias, 60% of studies were judged “high risk”, which was due to recruitment methods that used specific designs – common in clinical neuropsychological research – rather than a random or consecutive sampling procedure. Moreover, most studies relied on convenience samples recruited in clinical settings, which may limit the representativeness and generalizability of findings. For the index test and reference standard, all studies were rated as “low risk” because the administration of the tests was standardized. Regarding flow and timing, 20% had a “high risk of bias” and an “unclear risk of bias”: this was mainly because participants were excluded from analyses (i.e., patients completed test–retest sessions after the deadline). Although these aspects do not invalidate the present meta-analysis, they should be considered when interpreting the findings.

### Synthesis of results

**Fig. 2:**
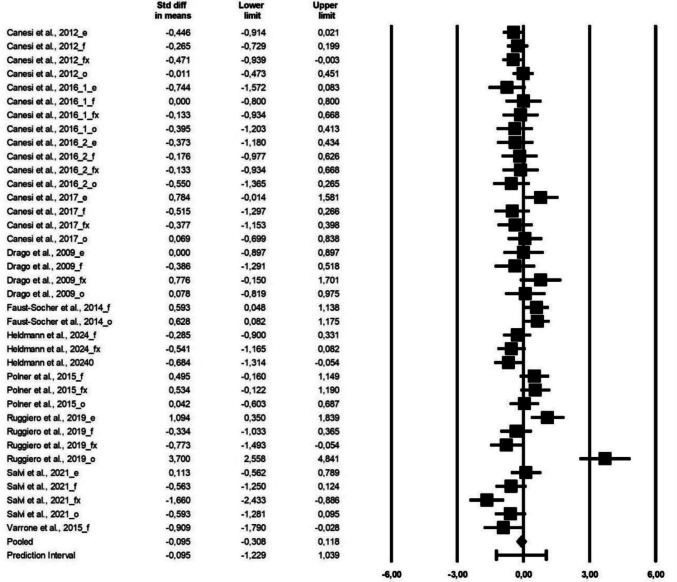
Forest plot of the divergent-thinking parameters. Legend: e: elaboration; f: fluidity; fx: flexibility; o: originality

The mean effect size is −0,095 with a 95% CI of −0,308 to 0,118, thus indicating an absence of significant differences between the two groups (see Fig. 2 for the Forest plot).

Analyzing the prediction interval, which reflects the dispersion effect, we can state that the true effect size in 95% of all comparable populations falls in the interval ranged from −1.229 to 1.039. From analyses of the heterogeneity, a high heterogeneity across the studies was confirmed: Q(df = 36) = 124.785, p < 0.001, *τ*^*2*^ = 0.3. Moreover, I^2^ = 71.150 highlighted that around 71% of the variance in observed effects reflects variance in true effects rather than sampling error [64]. Heterogeneity can have many clinical and methodological causes, such as the presence of participants’ different clinical features and differences in research designs [65, 66], such as the use of different tasks adopted to assess divergent thinking.

To verify sources of potential anomalies in the dataset, we performed a sensitivity analysis, recalculating the statistics with one study removed, in turn, for the entire distribution: no substantial differences emerged.

Subgroup analyses revealed mild, non-significant differences among the four parameters: overall: −0.126 (95% CI: −0.320, 0.067); prediction interval: −1.257, 1.004; Q(df = 3) = 2.476, p = 0.480. Specifically, fluency: −0.169 (95% CI: −0.439, 0.102), Q(df = 10) = 17.021, p = 0.074, *τ*^*2*^ = 0.084, I^2^ = 41.249; flexibility: −0.326 (95% CI: −0.752, 0.099), Q(df = 8) = 26.169, p = 0.001, *τ*^*2*^ = 0.287, I^2^ = 69.43; elaboration: 0.052 (95% CI: −0.441, 0.545), Q(df = 6) = 19.53, p = 0.003, *τ*^*2*^ = 0.299, I^2^ = 69.278; originality: 0.157 (95% CI: −0.389, 0.704), Q(df = 9) = 55.42, p < 0.001, *τ*^*2*^ = 0.633, I^2^ = 83.76; See Supplementary Fig. 1 (Online Resource) for the Forest plot for subgroup analyses. Therefore, we can state that, for most parameters, there is no significant and substantial difference between the PD patients and healthy controls, even if it is worth noting from subgroup analyses that patients presented mean lower performances than healthy controls in fluency and flexibility (even if not in a significant way) and patients presented a mean higher performance than healthy controls in originality [67]. Anyway, the large heterogeneity (especially for flexibility and originality) suggests that results should be considered with caution.

Meta-regression analyses with 26 cases were run using the Knapp-Hartung method (for further information, see: [68]). For each additional mg of medication, the effect size increased around by 0.0005 units (b = 0.0005, SE = 0.0002, t(24) = 2.04, p = 0.053, R^2^ = 0.11), indicating a mild but non-significant effect. The confidence interval at 95% ranged from < 0.001 to 0.001. Anyway, it is worth noting that these results should be considered with caution, as (i) they can be biased by other factors or confounding variables, as the severity and the duration of the disease, or age, and (ii) LEDDs are aggregated to the study level, even if they exhibit variations among every clinical sample of the studies considered. This may lead to a reduction in the precision of analyses.

**Fig. 3:**
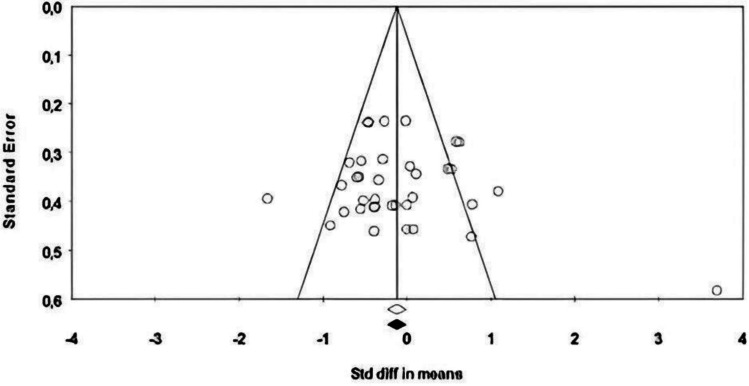
Funnel plot of the effect sizes of the considered studies

Lastly, the publication bias was assessed using the funnel plot, reporting no estimated numbers of missing studies to make the funnel plot symmetric (see Fig. 3). Using Trim and Fill these values are almost unchanged: under the random-effect model the point estimate was −0,09481 (95% CI: −0,30,798, 0,11,835).

## Discussion

The main aim of the present paper was to delve into possible differences in divergent thinking between PD patients and healthy controls by verifying the hypothesis that patients present higher levels of creative thinking through the analysis of the findings to date available in the literature.

From the analyses of the studies considered, it was highlighted an absence of statistically significant differences between PD patients and healthy control groups in the parameters usually employed to measure divergent-thinking tasks. This would mean that in general PD patients under dopaminergic treatment – when compared to healthy individuals – do not present significantly higher mean levels of divergent thinking, which is considered the cognitive ability to generate multiple ideas that are both original and useful and constitutes a core component of creative thinking.

The presence of single cases reported in literature and describing the onset of artistic activities in some patients after the beginning of the dopaminergic drugs would imply something different from the concept of creative thinking, as stated by Canesi and colleagues [42] when they discussed their results. A possible hypothesis, supported by the present findings, is that the production of artistic activities – such as paintings or written works – may not be identified with or mainly driven by cognitive processes related to divergent thinking (e.g., the ability to think in different directions and combine different domains to create something that is both original and with a useful value). Instead, it may also be influenced by other mechanisms, such as the intrinsic motivation and feeling of pleasure in making such activities or emotional rewards derived from the act of creating, where probably the mesolimbic dopaminergic pathways play a role [32, 33, 41].

Moreover, other individual differences can be involved in the artistic onset: Genetic predisposition (in particular, genes involved in dopamine transmission are under investigation) or personality traits can foster and support artistic expression [22, 35, 42, 43, 69, 70]. Socio-cultural and environmental factors may also influence divergent thinking, by providing physical and social stimulation. For instance, the exposure to multiple and conflicting value systems belonging to different cultures can foster greater cognitive flexibility and creative thinking [71]. Also the cultural system can influence the number and quality of generated idea (e.g., [72, 73]). Furthermore, few studies highlighted that living in small rural settings vs urban environments can lead to differences in parameters such as fluency, flexibility, and originality [74, 75]. In the studies considered for this paper, participants’ cultural backgrounds or potential cultural differences within the samples were not considered and, although these studies originate from various countries (USA, Israel, and Europe, with a predominance of studies from Italy), the control groups used for comparing divergent-thinking performance with PD patients were recruited in contexts similar to those experienced by the respective groups of patients. However, it would be valuable to explore the influence of such variables through future research, especially if considering that most of the studies investigating these influences are based on samples of children or students, with only a small portion focusing on adult or older samples.

Furthermore, we cannot exclude that the continuous practice in an artistic activity (when it aims at the final production of something that is appropriate or meaningful) may stimulate cognitive abilities that are at the basis of divergent thinking, such as focused and sustained attention, planning and monitoring, inhibition of inappropriate actions, cognitive flexibility, the ability to retrieve semantic or salient aspects from memory, and to connect distant aspects of reality. Consequently, the continuous and regular engagement in artistic activities can improve scores in divergent-thinking tasks, according to results from Canesi and colleagues [39, 42]. They compared the divergent-thinking performances of PD patients – who were not professional artists before the onset of the PD – dividing them into those who developed an artistic output after the introduction of dopaminergic medications and those who never showed artistic productions after the dopamine intake. The first subgroup reported higher scores than the second one in the elaboration parameter [39] and in the elaboration and originality parameters [42].

Moreover, as discussed later, it is worth keeping in mind that, although the analyses did not show significantly higher divergent thinking in patients, they also did not reveal lower levels compared to healthy controls. These findings may suggest that divergent thinking does not appear to be systematically impaired in PD patients so far. Although this should be interpreted cautiously, it may be hypothesized that some aspects of divergent thinking remain relatively maintained and could potentially represent a relevant area for future research and intervention in the field of cognition in PD.

Given the variability and inconsistency of findings in existing literature on this topic, we considered a meta-analytic approach necessary. It provides a quantitative synthesis of data across studies, allowing for the statistical integration of results and offering a more objective and transparent summary of the evidence, even when results are not statistically significant. This is the first study to adopt a meta-analytic approach to this issue, thus providing an updated overview of the literature and applying what is considered the gold standard for reliable and transparent reviews (e.g., [50]). This would offer a stronger empirical basis for clinicians and researchers involved in designing care pathways for PD patients, and more generally in cognitive functioning in PD.

Moreover, analyzing the studies considered, some important issues have emerged that warrant further investigation to fill the gaps that still exist in the literature and to gain a more detailed understanding of the topic. They are reported below.

### The role of cognitive abilities in divergent thinking

Focusing on the cognitive abilities required by creative thinking, it would be interesting to delve into possible relationships between the scores of divergent-thinking tasks and those of cognitive tests in PD patients. To date, a small number of studies have investigated possible relationships between cognitive functions and divergent thinking in PD. For instance, Canesi and colleagues [54], pooling all recruited groups for the study, found significant relationships between the ATTA total score and tests assessing executive functions (in particular phonemic fluency) and visuospatial abilities, but in other studies, when at least one cognitive function was investigated, it seems that the cognitive performance did not explain divergent-thinking performances ([52] concerning phonemic fluency; [57] for inhibition).

It would be important to assess whether and which cognitive abilities supposed to sustain creative thinking are related to divergent thinking in such patients, to better understand if creative thinking can support PD patients’ cognitive functioning, such as executive functions, preventing possible impairments that can bias their quality of life and autonomy. In fact, the results of the present analyses did not reveal significant higher mean levels of divergent thinking in patients, but it is true as well that they did not reveal significantly lower mean levels of divergent thinking compared to healthy controls. Thus, it is possible that this ability may remain relatively less affected than other cognitive functions. Accordingly, further studies may investigate whether divergent thinking could play a role in sustaining patients’ cognitive functioning, as it is highlighted in other studies investigating other neurological conditions and healthy aging [19, 76, 77]. In this way, Heldmann and colleagues [58] found significant lower performances of patients in a categorical fluency subtest and in an attentional subtest, but found that they obtained significant lower scores only in the originality parameter but not in fluency or flexibility. Salvi and colleagues [57] reported significantly lower performances by patients in some tests evaluating executive functions and immediate memory recall but found that they obtained significantly lower scores only in the flexibility parameter but not either in fluency, originality, and elaboration.

On the other hand, Varrone and colleagues [56] considered only the divergent-thinking parameter of fluency, which was lower in patients, and found significantly poorer performances in semantic and episodic memory in patients together with a trend toward significantly poorer working memory performance. It would have been interesting to examine whether patients also obtained significantly lower scores than healthy controls in other divergent-thinking parameters. Further studies are needed to delve into how divergent thinking is related to other cognitive abilities in PD patients, to deepen whether it can contribute to supporting cognitive functioning in such patients.

### The role of dopaminergic medications in divergent thinking

Another issue that deserves to be deepened is the possible role of dopaminergic medications in divergent-thinking performance, even if results from meta-regression were non-significant. Considering theoretical evidence in literature, it is possible that, at least in part, dopamine intake can influence divergent thinking in an indirect or complex way. Considering studies that explicitly reported to have examined possible relationships or a possible influence of dopaminergic medications (by considering the total LEDD) on divergent-thinking tasks, inconsistent results emerged. Canesi and colleagues [39, 42] reported the absence of significant relationships between divergent-thinking performances and LEDD, as well as Salvi and colleagues [57], displaying no differences in the divergent-thinking parameters when patients’ performances were compared during the “on” vs “off” conditions. Conversely, Faust-Socher and colleagues [43] found a positive correlation between LEDD and originality in the visual subtest of divergent thinking. Dividing patients into three groups according to the daily dose intake of medication, the high-LEDD group (dose = 442.82 ± 108.97 mg) reported a higher number of original responses compared to the low-LEDD group (dose = 148.53 ± 75.29 mg). Similarly, Heldmann and colleagues [58] highlighted a significant positive relationship between the originality parameter and both the total LEDD and when only the dosage of dopamine agonists was considered (18 out of 20 patients were receiving them). Ruggiero and colleagues’ [55] results are also worth mentioning, where a significant positive relationship was found between LEDD and the “T factor” that assessed the ability to produce relevant titles for each drawing produced. Such a parameter, involving the complexity of the vocabulary adopted, may require semantic and linguistic skills, in addition to unusual ways to combine linguistic terms. Thus, it seems that when significant correlations emerged between divergent-thinking parameters and LEDD, they involved originality.

This can be consistent with the assumptions stating that dopaminergic medications can support creative production by lowering latent inhibition, and so facilitating access to more remote associations and encouraging the original recombination of ideas (e.g., [32, 34]). However, the issue appears more complex when considering the results of a study that compared divergent-thinking performance in de novo PD patients at baseline (before starting the dopaminergic therapy) and 12 weeks after [62]. Although improvements were observed in fluency and flexibility parameters following treatment, originality did not increase significantly. Further studies would be useful to better understand the relationships between dopaminergic medications and divergent-thinking performances, also to deepen the effect of striatal dopamine on flexible thinking [32].

Moreover, it can be useful to focus not only on investigating the relationships in question by considering the total LEDD, but also by calculating the LEDD for each dopaminergic medication (e.g., levodopa, dopamine agonists), as it was done by some studies with inconsistent results (e.g., [54, 58]). Different dopaminergic medications may exert partially distinct effects on cognition, which may be hidden when considering only the total LEDD. This may reflect the differential modulation of dopaminergic pathways and frontostriatal circuits involved in reward-related processing, novelty seeking, and cognitive flexibility, as previously highlighted in studies investigating other cognitive abilities (e.g., [13, 78]) and proposed in models of creative cognition [32, 33, 79]. However, more detailed quantitative analyses were not feasible in the present meta-analysis because only a limited number of studies reported medication-specific LEDD data, most often for dopamine agonists only [42, 54, 58].

### The divergent-thinking assessment

It is also worth mentioning that the studies considered adopted different tasks to assess divergent thinking, as reported in Table 2. Although all tasks were designed to measure divergent thinking – and therefore required the generation of multiple ideas or solutions – we cannot exclude that, at least in part, they differed in their specific cognitive and behavioral demands. For instance, the AUT, the ATTA, the subtests of the TTCT, and the DTT may engage partially distinct processes, relying on different cognitive abilities, such as verbal fluency, semantic retrieval, cognitive flexibility, inhibition, visuospatial abilities, planning, and motor output. These functions may be differentially affected in PD, potentially contributing to variability across findings. Therefore, methodological differences between creativity measures may have attenuated potential effects and reduced the comparability of results across studies.

At the same time, it should also be considered that several studies relied on partially overlapping paradigms. Three studies adopted the ATTA [42, 52, 54] and four the AUT [43, 56–58]. Similarly, the verbal “Just Suppose” TTCT subtest, requiring participants to imagine the consequences of an unrealistic scenario, may conceptually partially overlap with one of the ATTA activities, involving the generation of consequences from hypothetical situations. Likewise, the DTT, based on the repeated use of a simple geometric figure to create different drawings, may partially resemble one of the visual activities included in the ATTA. Therefore, these results should be interpreted taking these aspects into account.

More generally, this heterogeneity may also reflect the relatively recent and still evolving interest in divergent thinking within neuropsychological research, particularly when compared to more extensively investigated domains such as memory, attention, or executive functions. In this regard, future research may benefit from the development and wider adoption of more standardized assessment approaches, potentially allowing greater comparability across studies.

### Limitations

The present paper presents some limitations. First of all, the strict inclusion criteria, aimed at reducing heterogeneity among the included studies ensuring the comparability of results, have limited the number of studies considered. In this way, we decided to focus our attention on divergent thinking which is considered a proxy of creative potential [44], without considering convergent-thinking measures. This choice was motivated by the intention to reduce conceptual and methodological heterogeneity, as convergent thinking is conceptualized as the other type of creative process and entails the finding of a single solution to a well-defined problem by restructuring and linking concepts previously known [22]. Although related, divergent and convergent thinking may rely on different neurocognitive mechanisms (e.g., [22]) and may also be differentially affected in PD. Secondly, the unavoidable heterogeneity of the studies – due to differences in the size of the recruited samples, sociodemographic and clinical characteristics of patients, different tasks used to assess divergent thinking (as previously discussed), and research designs – suggests that results should be considered with caution. In this regard, a random-effects model was adopted in the meta-analysis to account for, at least in part, the expected variability across studies. Nevertheless, the variability in methodological approaches and clinical characteristics across the included studies may have contributed to masking differences that could emerge in specific experimental or clinical contexts. More specifically, it is worth noting that patients considered in the presented studies belonged to different stages of the disease and had different disease durations (see Table 1); Patients at more advanced stages of PD can display a higher cortico-subcortical impairment compared with those that were at the initial stages of the disease, potentially affecting cognitive performance more substantially. We cannot exclude that they may represent potential confounding factors influencing divergent-thinking performance. At the same time, even if the presence of severe cognitive impairments was an exclusion criterion for being enrolled in the studies considered, we cannot exclude the presence in some participants of subtle alterations in cognitive abilities potentially relevant to divergent thinking. As it was previously discussed, further research would be crucial to shed light on the relationships between specific cognitive abilities and divergent-thinking performance.

## Conclusions

Considering the presence of conflicting results in research that delved into divergent thinking in PD by comparing patients regularly assuming dopaminergic medications and healthy controls, a statistical synthesis of data present in the literature was needed, to achieve a broader and comprehensive understanding of the issue. Findings suggest that divergent-thinking abilities may not be impaired by the disease (as no statistically significant difference in the average score was found between patients and healthy controls). Although this interpretation should be considered cautiously, future studies may explore whether some aspects of divergent thinking could potentially represent a relevant area for supporting cognitive functioning in PD. In fact, creative and divergent thinking underlie a plethora of cognitive abilities, such as executive functioning and memory retrieval, that are pivotal not only for optimal cognitive functioning but also for coping with everyday-life situations. For instance, it is highlighted that impairments in executive functions – that are linked to higher probabilities of developing dementia in PD (e.g., [80]) – can lead patients to replace functional behaviors to solve obstacles and problems in daily situations with others that are less functional and more stereotyped, which may be inappropriate for the demand [81]. Moreover, there is evidence supporting that executive functions (in particular flexibility) and memory are associated with the ability to understand and implement medication management in PD patients – which can be complex till the early stages of the disease – and even mild impairments in such abilities can bias the medication adherence, leading to negative clinical consequences [82]. Thus, it appears crucial for patients’ wellbeing to keep the focus on possible ways to effectively prevent impairments, and cognitively fostering divergent thinking may be useful. Accordingly, preliminary findings with other neurological targets showed that adopting cognitive training underlying divergent thinking in patients with focal brain lesions in the chronic phase (as they were recruited at least one year after the stroke) can improve cognitive abilities such as executive functions and memory [76]. Moreover, as creative thinking is assumed to rely on motivational processes supported by the mesolimbic circuit [33, 41], the proposal of exercises based on divergent thinking can make the scheduled activities motivating and engaging, possibly increasing the level of effort exerted by the patient.

Furthermore, as the literature highlights the potential of divergent-thinking tasks in detecting possible early cognitive changes in patients with a diagnosis of MCI or mild dementia of the Alzheimer’s or frontotemporal type [77, 83], further studies would be useful to investigate if these tasks can be sensitive also for detecting early cognitive difficulties in PD patients. This could be useful for designing tailored clinical pathways for patients, aimed at slowing down the cognitive decline and supporting the wellbeing of both patients and their caregivers.

## Supplementary Information

Below is the link to the electronic supplementary material.

Supplementary file1 (PDF 309 KB)

## Data Availability

The dataset generated and analysed during the current study is available in the OSF repository, https://osf.io/km3j6/?view_only=d01f4b2436ba4105970505e027214420. The review protocol was registered in PROSPERO: CRD42022319590, https://www.crd.york.ac.uk/prospero/display_record.php?RecordID=319590. Other data that support the findings of this study are available on request from the corresponding author.
