# Supplementary material for: Creative thinking in Parkinson’s disease: A systematic review and meta-analysis
Source: Neurol Sci. 2026 Jul 16;47(8):632. doi: 10.1007/s10072-026-09234-7 (PMC13372961; doi:10.1007/s10072-026-09234-7)

Supplementary file

Supplementary Table 1 Further information for data considered

Study	Data format for divergent thinking parameters	Groups considered	Divergent-thinking parameters
Drago et al., 2009	Independent groups (means, sd)	15 pooled PD patients (composed by either right or left onset PD) and 7 healthy controls	fluency, flexibility, elaboration, originality
Canesi et al., 2012	Independent groups (means, sd)	36 pooled PD patients (composed by patients either with increased artistic production and without increased artistic production) and 36 healthy controls	fluency, flexibility, elaboration, originality
Faust-Socher et al., 2014	Independent groups (difference, p-value)	27 PD patients and 27 healthy controls	fluency, originality
Varrone et al., 2015	Independent groups (means, sd)	10 PD patients and 10 healthy controls	fluency
Polner et al., 2015	Independent groups (means, sd)	18 PD patients (considering scores obtained from the follow-up session, where patients were evaluated for the second time 12 weeks after starting the dopaminergic therapy) and 19 healthy controls	fluency, flexibility, originality
Canesi et al., 2016	Independent groups (means, sd)	12 PD patients professional artists before the onset of PD and 12 healthy controls professional artists [Canesi et al., 2016_1]; 12 PD patients who never showed artistic productions and 12 healthy controls who never presented creative outputs [Canesi et al., 2016_2]; we did not include in the analyses the group composed of 12 PD patients separately assessed, who were not professional artists before the onset of the disease and developed artistic output after the onset of PD and the introduction of the dopaminergic treatment, as they were mainly cited in the article to compare their divergent-thining performance to those obtained from the other two clinical groups	fluency, flexibility, elaboration, originality
Canesi et al., 2017	Independent groups (means, sd)	13 PD patients and 13 healthy controls	fluency, flexibility, elaboration, originality
Ruggiero et al., 2019	Independent groups (means, sd)	17 PD patients and 15 healthy controls	fluency, flexibility, elaboration, originality

Salvi et al., 2021	Independent groups (means, sd)	13 PD patients (considering scores obtained from the assessment performed during the pharmacological "on" condition) and 26 healthy controls. In the study design, for PD patients two assessment sessions were scheduled, one during the "on" and one during"off" pharmacological condition, within a two-week interval and where patients were randomly assigned to one of two test-retest orders (ON-OFF: 6 patients,OFF-ON: 7 patients)	fluency, flexibility, elaboration, originality
Heldmann et al., 2024	Independent groups (difference, p-value)	20 PD patients and 20 healthy controls	fluency, flexibility, originality

Note: PD: Parkinson's disease; sd: standard deviation.

Supplementary Table 2 QUADAS-2 quality assessment of the included studies

Study	Risk of bias				Applicability Concerns	
	Patient selection	Index Test	Reference Standard	Flow and Timing	Patient selection	Reference Standard
Drago et al., 2009	U	L	L	L	L	L
Canesi et al., 2012	H	L	L	L	L	L
Faust-Socher et al., 2014	L	L	L	L	L	L
Varrone et al., 2015	H	L	L	H	L	L
Polner et al., 2015	H	L	L	L	L	L
Canesi et al., 2017	L	L	L	U	L	L
Canesi et al., 2016	L	L	L	U	L	L
Ruggiero et al., 2019	H	L	L	L	L	L
Salvi et al., 2021	H	L	L	H	L	L
Heldmann et al., 2024	H	L	L	L	L	L

H= high, L= low, U=unclear

Figures

Supplementary Fig. 1 Forest plot for subgroup analyses

Parameter		Std diff in means	Lower limit	Upper limit
elaboration	Canesi et al., 2012_e	-0,446	-0,914	0,021
elaboration	Canesi et al., 2016_1_e	-0,744	-1,572	0,083
elaboration	Canesi et al., 2016_2_e	-0,373	-1,180	0,434
elaboration	Canesi et al., 2017_e	0,784	-0,014	1,581
elaboration	Drago et al., 2009_e	0,000	-0,897	0,897
elaboration	Ruggiero et al., 2019_e	1,094	0,350	1,839
elaboration	Salvi et al., 2021_e	0,113	-0,562	0,789
elaboration	Pooled	0,052	-0,441	0,545
elaboration	Prediction Interval	0,052	-1,496	1,599
flexibility	Canesi et al., 2012_fx	-0,471	-0,939	-0,003
flexibility	Canesi et al., 2016_1_fx	-0,133	-0,934	0,668
flexibility	Canesi et al., 2016_2_fx	-0,133	-0,934	0,668
flexibility	Canesi et al., 2017_fx	-0,377	-1,153	0,398
flexibility	Drago et al., 2009_fx	0,776	-0,150	1,701
flexibility	Heldmann et al., 2024_fx	-0,541	-1,165	0,082
flexibility	Polner et al., 2015_fx	0,534	-0,122	1,190
flexibility	Ruggiero et al., 2019_fx	-0,773	-1,493	-0,054
flexibility	Salvi et al., 2021_fx	-1,660	-2,433	-0,886
flexibility	Pooled	-0,326	-0,752	0,099
flexibility	Prediction Interval	-0,326	-1,694	1,041
fluency	Canesi et al., 2012_f	-0,265	-0,729	0,199
fluency	Canesi et al., 2016_1_f	0,000	-0,800	0,800
fluency	Canesi et al., 2016_2_f	-0,176	-0,977	0,626
fluency	Canesi et al., 2017_f	-0,515	-1,297	0,266
fluency	Drago et al., 2009_f	-0,386	-1,291	0,518
fluency	Faust-Socher et al., 2014_f	0,593	0,048	1,138
fluency	Heldmann et al., 2024_f	-0,285	-0,900	0,331
fluency	Polner et al., 2015_f	0,495	-0,160	1,149
fluency	Ruggiero et al., 2019_f	-0,334	-1,033	0,365
fluency	Salvi et al., 2021_f	-0,563	-1,250	0,124
fluency	Varrone et al., 2015_f	-0,909	-1,790	-0,028
fluency	Pooled	-0,169	-0,439	0,102
fluency	Prediction Interval	-0,169	-0,895	0,557
originality	Canesi et al., 2012_o	-0,011	-0,473	0,451
originality	Canesi et al., 2016_1_o	-0,395	-1,203	0,413
originality	Canesi et al., 2016_2_o	-0,550	-1,365	0,265
originality	Canesi et al., 2017_o	0,069	-0,699	0,838
originality	Drago et al., 2009_o	0,078	-0,819	0,975
originality	Faust-Socher et al., 2014_o	0,628	0,082	1,175
originality	Heldmann et al., 20240	-0,684	-1,314	-0,054
originality	Polner et al., 2015_o	0,042	-0,603	0,687
originality	Ruggiero et al., 2019_o	3,700	2,558	4,841
originality	Salvi et al., 2021_o	-0,593	-1,281	0,095
originality	Pooled	0,157	-0,389	0,704
originality	Prediction Interval	0,157	-1,787	2,102
Overall	Pooled	-0,126	-0,320	0,067
Overall	Prediction Interval	-0,126	-1,257	1,004

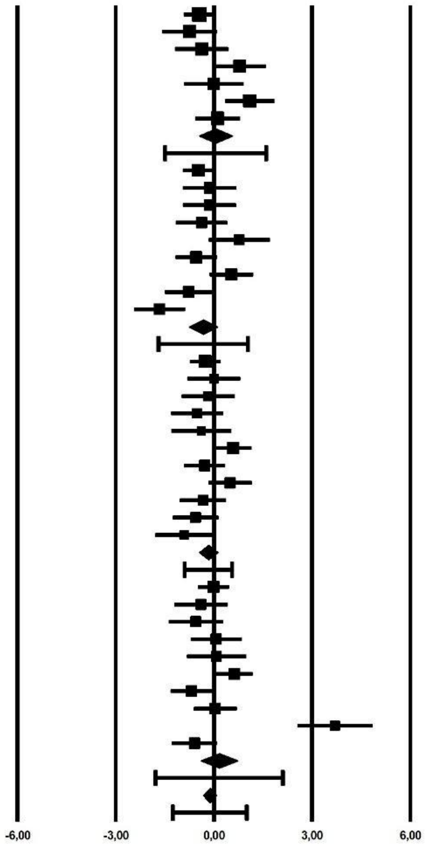

Supplement: Supplementary file 1 — Supplementary file1 (PDF 309 KB) [file 10072_2026_9234_MOESM1_ESM.pdf]
